# In the light of change: a mixed methods investigation of climate perceptions and the instrumental record in northern Sweden

**DOI:** 10.1007/s11111-018-0302-x

**Published:** 2018-08-28

**Authors:** Maria Furberg, David M. Hondula, Michael V. Saha, Maria Nilsson

**Affiliations:** 1 Department of Public Health and Clinical Medicine, Epidemiology and Global Health, Umeå University, SE-901 87 Umeå, Sweden; 2 Department of Clinical Microbiology, Infectious Diseases, Umeå University, SE-901 85 Umeå, Sweden; 3 School of Geographical Sciences and Urban Planning, Arizona State University, Tempe, AZ 85287 USA; 4 Department of Environmental Sciences, University of Virginia, Charlottesville, VA 22904 USA

**Keywords:** Climate change, Indigenous peoples, Mixed methods, Reindeer herding, Cold spells, Variability

## Abstract

Significant climate change in the Arctic has been observed by indigenous peoples and reported in scientific literature, but there has been little research comparing these two knowledge bases. In this study, Sami reindeer herder interviews and observational weather data were combined to provide a comprehensive description of climate changes in Northern Sweden. The interviewees described warmer winters, shorter snow seasons and cold periods, and increased temperature variability. Weather data supported three of these four observed changes; the only change not evident in the weather data was increased temperature variability. Winter temperatures increased, the number of days in cold periods was significantly reduced, and some stations displayed a 2 month-shorter snow cover season. Interviewees reported that these changes to the wintertime climate are significant, impact their identity, and threaten their livelihood. If consistency between human observations of changing weather patterns and the instrumental meteorological record is observed elsewhere, mixed methods research like this study can produce a clearer, more societally relevant understanding of how the climate is changing and the impacts of those changes on human well-being.

**Electronic supplementary material:**

The online version of this article (10.1007/s11111-018-0302-x) contains supplementary material, which is available to authorized users.

## Introduction

Climate change is affecting the world in many ways at different speeds in different places. Some of the most rapid changes are taking place in the Arctic, including rising air temperatures and decreasing extent of sea ice (IPCC 2013). Rapid climate change in the Arctic is affecting local populations, including indigenous peoples, who started noticing changes at an early stage, several decades ago (Ashford & Casteldon 2001; Krupnik & Jolly 2002).

Beyond the quantitative approach that is characteristic of much climate change research, qualitative methods can bring important perspectives and findings to light on the subject. Qualitative methods are particularly well suited for investigating local perceptions of climate change, which can yield insights into gaps between public understanding and quantitative climate trends reported by physical scientists. Understanding these perceptions is important as they drive behaviors and inform policymaking (Weber 2010). Policy changes associated with climate change are and will likely need to be extensive and far-reaching to minimize risks to human society and ecosystems (Watts et al. 2015).

The physical science enterprise is investing extensive resources to monitor and model climate change, especially at regional to global scales. Policy makers have relied heavily on observations and models of the physical system at these scales to craft a primarily top-down communication strategy about climate change and its potential impacts (Ockwell et al. 2009; European Commission 2014). But climate change effects on weather, environment, and people are complex; these effects span objective as well as subjective phenomena characterized by great variability on global, regional, local, and individual levels. There are many localities, especially in developing countries, lacking sufficient meteorological instrumentation to assess long-term climatic trends. Even in places where long-term meteorological records do exist, scholars have so far not well-documented the association between climatological data and perceptions of people experiencing climate change. Mixed methods play an important role in filling that gap, but the promise of interdisciplinary climate change research has been realized far too infrequently, despite repeated efforts to highlight its potential (Costello et al. 2009; Kathleen Ann & Petra 2010; Riedlinger & Berkes 2001).

There is a small but growing literature using qualitative and quantitative methods together in the context of climate change in an attempt to complement and further elaborate upon the results obtained from each method (Doyle et al. 2013). Only a few published studies within the climate change research field formally embody mixed methods research, in which researchers draw meta-inferences from the combination of the two methods, rather than just reporting the two different sets of results (Cuerrier et al. 2015; Cunsolo Willox et al. 2012; Gearheard et al. 2010; Marin, 2010). The lack of a rich mixed methods literature in climate change research, especially concerning indigenous peoples, may be attributable to the inherent challenges of integrating qualitative and quantitative methods across multiple disciplines. The deficiency is unfortunately reflective of the more widespread underrepresentation of local communities and traditional knowledge in climate science and policy discourse (Smith and Sharp 2012; Ford et al. 2016).

The magnitude of the historical and future changes in the Arctic climate, related impacts on the Sami and other regional populations, and increasing awareness of the role of perception in climate-related policy and practice motivated us to combine local perceptions and meteorological instrumental record data in this mixed methods study. The level of interaction between the different data sets in this study is interactive: the quantitative analyses were designed based on phenomena identified in the qualitative interviews. The quantitative data were analyzed separately before the final merged interpretation took place (Creswell 2011). The strength of this dependent sequential design is that the subsequent quantitative strand provides a more objective scale for evaluation of the changes described in the qualitative data. The study design also allows regional generalization of participant perceptions beyond the reindeer herding society, further increases validity of the qualitative results, and enables the detection of emergent theories through the discovery of paradoxes or contradictions. The major disadvantages of the design are its complexity, time and resource consumption, and the difficulty in resolving discrepancies that arise when interpreting the findings.

Insights gained from this distinctive combination of climatological observational data and local perceptions could contribute to more evidence-driven policy making concerning climate change in the Arctic region of Sweden.

## Background

The qualitative study was originally performed to get a view of peoples’ experiences and perceptions of climate change in northern Sweden (Furberg et al. 2011). The reindeer herders’ sensitivity to environmental changes primarily stems from their subsistence lifestyle with total dependence on the available grazing lands for the reindeer. Traditional lifestyles are based on shared and inherited experiences and traditional knowledge gained over generations. The Sami are the Swedish indigenous population and, in contrast to many other indigenous peoples of the Arctic, the majority of Sami are completely integrated into the Swedish society secondary to centuries of internal colonization. In the colonization process, the Sami lost control and authority over the land and Sami culture, identity and rights were tied exclusively to reindeer herding on which regulations were imposed by the government (Brannlund & Axelsson 2011). The friction inflicted by this colonial heritage is still affecting the Sami society and the relationship between the Sami people, particularly the reindeer herders, and parts of the major society (Nordin 2002; Kaiser et al. 2010).

The available research on Sami health in Sweden suggests that the Sami populations’ overall health status equals that of the general Swedish population in terms of major diseases, causes of death, and overall life expectancy. Educational level, occupation, and socioeconomic standards are also equivalent for the majority of the Sami population when compared to the whole of Sweden (Hassler et al. 2005, 2008). Yet, integration has occurred at a cost of cultural identity and connection to the traditional Sami land and lifestyle. The number of reindeer in Sweden have varied between 150,000 and 300,000 in the last 100 years with the latest peak in 1990, the fluctuations due to variations in grazing and predator pressure (Jernsletten & Klokov 2002). According to the Swedish Sami Parliament (2018), about 2500 herders in Sweden have their main income from reindeer herding, of whom about 18% are women.

Reindeer herding is based entirely on the sustainable and free exploitation of natural pastures, which is regulated by Swedish law. It is a regional activity with annual migrations twice a year along a northwest-southeast axis for mountain Sami villages, between the summer grazing lands in the mountains to the winter pastures closer to the coast. Migration is traditionally made on foot, for some a distance of up to 300 km, and the herders accompany the herd on foot, skis, snowmobiles, and motorcycles. For this reason, in the present study, the herders’ experiences and perceptions of weather and climate are considered regional as well as local in character and scale.

## Materials and methods

An exploratory sequential mixed methods study was performed where the initial qualitative study was followed by the creation of meteorological variables tested with quantitative methods (Creswell 2011). Phenomena that greatly impact reindeer herding activities and were described or emphasized by several interviewees were chosen for further elaboration and testing.

The hypotheses for the mixed methods study were that local perceptions, especially those of indigenous peoples leading traditional lifestyles, are highly sensitive to climate change effects and therefore will be supported by meteorological instrumental records. Local perceptions might also discover ongoing changes in climate hitherto not reported, recognized or studied.

### Initial qualitative study

**Fig. 1:**
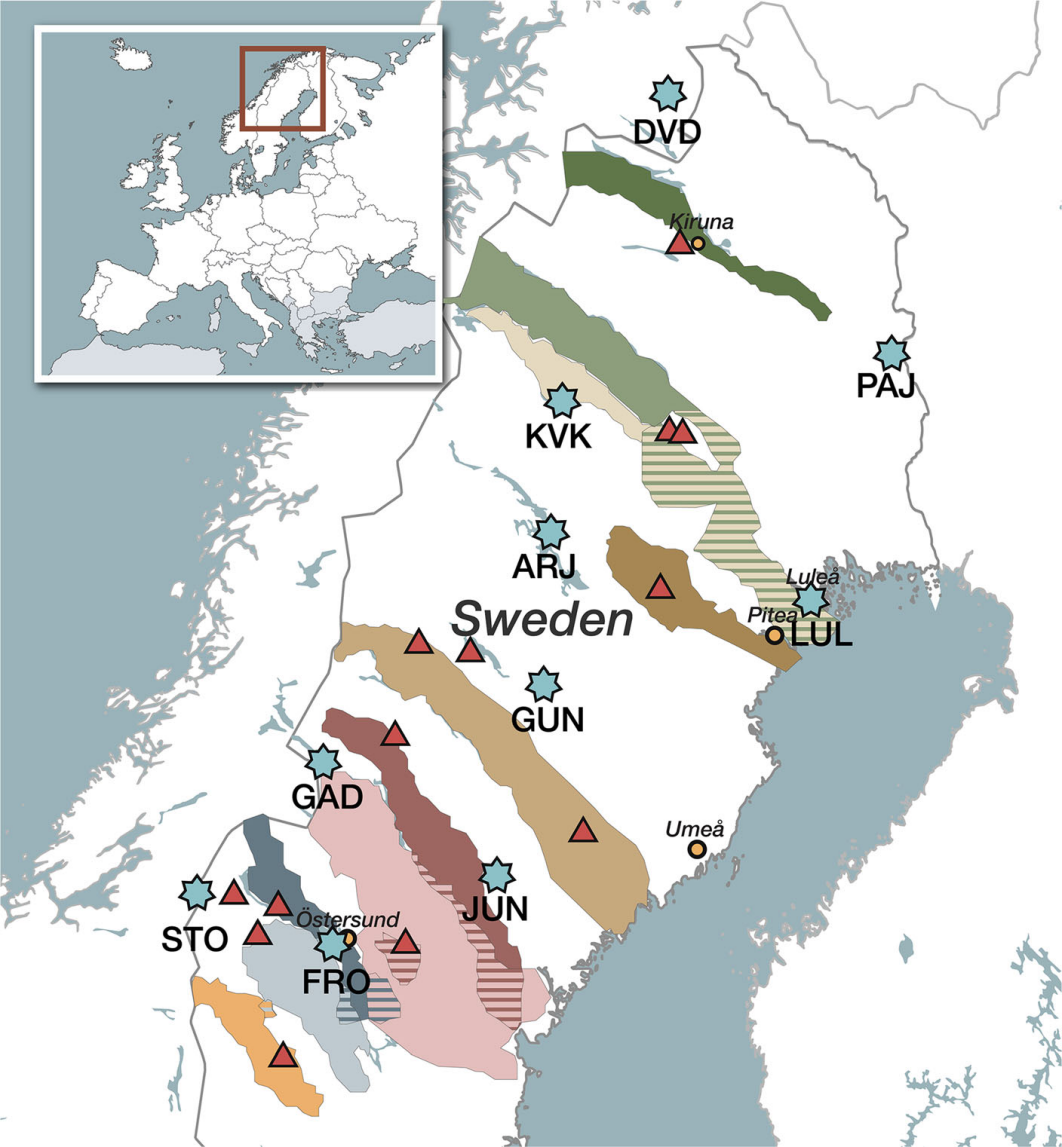
Interviewees’ residences (*triangles*), Sami village and grazing areas (*shaded areas*), and weather stations (*stars*). Cities of Ostersund, Umea, Pitea, and Kiruna marked as reference points. Striped areas mark shared grazing lands. Illustration by www.nopolo.se

The initial study phase was qualitative with recorded informal in-depth interviews with 14 reindeer herders examining their lived experiences of changes in the environment. Sami reindeer herders were chosen through purposive sampling as local northern Sweden population representatives since they were considered the most knowledgeable in the context of climate and weather variability. The study received the consent of the Sami Parliament and approval of the Research Ethics Committee at Umea University (dnr 09_193 § 48/09). All interviews were performed by the first author who is non-Sami, brought up in northern Sweden alongside the Sami reindeer herding society, but without personal involvement with the lifestyle. The study aimed at maximum variation sampling and the interviewees were 3 women and 11 men, 16–75 years of age; geographically dispersed (Fig. 1) with an average of 39 years of reindeer herding experience. A thematic interview guide was used and respondents were asked open-ended questions about experiences of changes to the environment, what they thought about these changes and the future for reindeer herding. The number of interviews proceeded until the data appeared saturated. All interviews were performed during spring 2010, mainly in respondents’ own homes.

The interviews were transcribed verbatim and analyzed using qualitative content analysis. This analysis involves identifying meaningful units to code and sort the data into subcategories, followed by the sorting of ideas generated by the data thereby moving the analyses to a higher interpretive level. Subcategories are merged into categories and one or several core themes emerge (Graneheim & Lundman 2004; Krippendorff 2004). Peer review processes were used during coding procedures as well as during analyses for triangulation purposes and to increase trustworthiness and validity.

In the present mixed methods study, only 13 interviews were used in the analyses, making the mean and median age of the participants 55 and 56 years, respectively. The discarded interview (one woman) did not match the current geographical study area.

### Creation of variables for the subsequent quantitative strand

The variables for the quantitative study were chosen based on reported changes that have a great impact on the reindeer herders’ daily life and work, implicating a possibly even higher sensitivity to these specific changes. A number of quantitative measurements of climate variability in northern Sweden over the past three decades were selected to visualize the regional trends and to compare instrumental records to respondents’ perceptions. Four specific observations (O1–O4) from the reindeer herding Sami identified in the interview study were adopted for the subsequent quantitative investigation:

- O1: The snow season is beginning later and ending sooner.
- O2: Wintertime temperatures are increasing.
- O3: Long stable cold periods are becoming less common.
- O4: Rapid fluctuations in temperature are becoming more common.

### Quantitative investigation methods

Daily measurements of maximum and minimum temperature and snow depth were obtained for 10 stations in northern Sweden and northern Norway (Fig. 1). Stations were selected based on data availability and proximity to the residences of the interview study subjects. Since the period of record for each station varies, data from the period 1978–2007 were used, consistent with the relevant time scale of the interviews (Table 1). These quality-controlled meteorological data were downloaded from the US National Climatic Data Center, accessible via http://www.ncdc.noaa.gov. Separate quantitative analyses were conducted to test each of the Sami observations listed above (O1–O4). The quantitative methods are detailed below. Statistical analyses and graphics were completed in MATLAB version 7.10.0 (The MathWorks, Inc., USA) and R version 2.13.2.

- O1: The snow season is beginning later and ending sooner.

**Table 1:** Geographic details for the meteorological stations used in this study. Symbols used to represent each station on the map are shown in the first column. The period of record for all stations is 1978–2007

Symbol	Name	Longitude (°E)	Latitude (°N)	Elevation (m)
ARJ	Arjeplog	17.84	66.05	431
DVD	Dividalen	19.71	68.78	228
FRO	Frösön	14.49	63.20	376
GAD	Gaddede	14.16	64.5	328
GUN	Gunnarn	17.71	65.01	280
JUN	Junsele	16.95	63.68	215
KVK	Kvikkjokk	18.02	66.89	314
LUL	Lulea	22.12	65.54	17
PAJ	Pajala	23.39	67.21	168
STO	Storlien	12.13	63.30	642

A 21-day-centered moving average smoother to the daily snow depth data was applied and each “snow season” was identified as any period of at least 50 consecutive days in which the smoothed snow depth time series was greater than 25 mm. The moving average was employed to exclude early and late season snowfalls that may precede or follow days with no snow cover, instead focusing on the period of the year in which snow depth consistently was above 25 mm. The snow depth was selected as an indicator for the minimum conditions in which the reindeer herders are able to use skis and snowmobiles when herding. The start date, end date, and duration of the snow season were calculated for each year, and the yearly time series tested for linear trends in each variable. This analysis spanned 28 full winters beginning with winter 1978/1979 and ending in winter 2006/2007.

- O2: Wintertime temperatures are increasing.

To determine how wintertime temperatures have changed, linear trends for all percentiles of minimum and maximum temperature were calculated on a station-by-station basis. A percentile-based approach was applied because various physical processes and social activities (and thus, perceptions) may be sensitive to different portions of the temperature distribution (Robeson 2004). Percentiles of minimum and maximum temperature were calculated on an annual basis for each station, excluding any years with gaps of missing data of 2 weeks or more. Least-squares linear regression was then used to examine the trend in each percentile of temperature at each station. In total, 2000 regression slopes and intercepts were calculated (100 per station × 10 stations, maximum and minimum temperature). Trends with *p* values less than 0.05 were considered significant.

- O3: Long stable cold periods are becoming less common.

The “cold wave” definition adopted in some temperature health research was employed (Barnett et al. 2011), where a period is defined as one of several days in which temperatures are consistently below a certain value. Daily mean temperature (*T*_MEAN_) was calculated as the average of the minimum and maximum daily temperatures. Cold periods were identified as those in which *T*_MEAN_ did not exceed the annual 25th percentile of *T*_MEAN_ for at least 10 consecutive days. The total number of cold period days within each year of record was then calculated and a quasi-Poisson regression was fitted to the time series of yearly counts. Sensitivity analysis was performed to test other percentile and duration thresholds.

- O4: Rapid fluctuations in temperature are becoming more common.

Three different approaches for assessing if the frequency of “rapid fluctuations” in temperature had changed over the time period were employed. First, trends in the percentiles of daily changes in maximum and minimum temperatures were examined using the regression method presented for O2, but using the variables Δ*T*_MAX_ and Δ*T*_MIN_, calculated as *T*_dayN_ − *T*_dayN-1_. To facilitate the possibility that the perceived changes in temperature fluctuation might occur over longer time scales, the temperature range for overlapping 5-day periods was also examined. The range for each period was calculated as the highest maximum temperature minus the lowest minimum temperature. The median 5-day temperature range for each month over the period of record along with the 75th, 90th, and 99th percentiles were calculated and linear regression was used to test for significant trends for each percentile and month. Finally, because the condition of the ground and standing water as frozen (or not) is easily observable by—and important to—the herders, trends in the number of days with daily maximum temperatures above freezing and daily maximum temperatures below zero were also investigated. Trends in the frequency of these “near freezing” days might be indicative of conditions that the herders associate with variation or change in the weather and/or unpredictability. At each station, the number of days each year with *T*_MAX_ above 1, 2, and 3 °C were tabulated coincident with days with *T*_MIN_ below − 1, − 2, and − 3 °C, respectively, and we assessed trends over time with quasi-Poisson regression.

## Results

### Qualitative study results

In general, the qualitative study concluded that climate change was just one more stressor that, added to multiple other stressors, were pushing the Sami reindeer herders toward the limit of their abilities to adapt to an evolving physical, social, cultural, ecological, and economic environment. Some respondents even indicated uncertainty about the viability of the reindeer herding lifestyle beyond the present generation. The already present stressors included tourism, hydropower, wind power, bad economic viability, constantly decreasing grazing lands, forestry, the building of new roads, and the very important factor of predator pressure and governmental predator policy,^1^ among others.

With respect to environmental changes, respondents identified changes in several different ways and they reported extensive alterations to the weather and climate since the 1970s, which they also perceived to have accelerated during the first decade of the 2000s. The changes included long and warm autumns, later onset of freezing conditions, shorter snow cover season, early and sudden onset of spring and more unstable, unpredictable and variable weather; each of these perceptions is described in more detail below.

*Autumns* were reported as longer, warmer, and wetter where persistent snow cover is established later in the season and water freezes over much later as well. This affects the reindeer herders’ ability to move the herd on foot during the annual migrations. Temperatures that persist around 0 °C during a rainy autumn can “lock” the grazing under a layer of ice instead of snow preventing the reindeer from accessing the food, thereby ruining the pasture for a whole winter under worst circumstances.

*When we moved down in the past, the waters froze in the autumn, there was a bit of snow, it was cold, it started in October already. Waters froze over in October and we used to move the herd one month before Christmas using all the lakes … and back then there were ice on them at that time… But now they stay open … until … well they are still open. -- herder 3*

*Rain and snow have started to come indiscriminately with the consequence that the grazing gets locked … and then the reindeer herds spread out ... and this favor the predators. -- herder 10*

*Winters* were experienced as much warmer and the long, cold, stable periods once familiar to the herders are more unusual or completely gone. These periods used to make it possible for herders to do other things knowing they did not have to be prepared to move the herd every day since the conditions were stable for a long period. The general impression among the herders was that the snow cover season had grown much shorter. The stories included some contradictory opinions regarding the end of the snow season though, especially in the high mountains where the snow cover was perceived to persist a lot longer than previously. Both of these contradictory observations were reported from the same area of Sarek national park (corresponding to station Kvikkjokk).

*The cold period became very short, when the temperatures went below 25 degrees, it was only for a few days … that is new … Last cold winter was in 1986. … From 1986 and onward, it got a lot warmer. We rarely had temperatures below 20 degrees … before then, winter temperatures reached minus 30- 35- 40 degrees Celsius. -- herder 10*

*There was snow in November and it used to be thaw around Epiphany and then cold lasted until spring but today weather is more helter-skelter. -- herder 10*

*Spring* was said to now come early and very abruptly, weeks earlier in many places than in the past. The perspective also existed that spring might then come to a halt for many weeks, making the winter pastures perishable and unfitting when the summer pastures are still inappropriate due to rough conditions and heavy snow. The period between winter and summer grazing has always been a difficult time and today that difficulty is exaggerated by the extensive forestry that has reduced old intact forests with arboreal beard lichen (*Usnea* of the family *Parmeliaceae*) to a minimum, beard lichen constituting the classic reindeer emergency forage.

*And already in March spring starts and in April with bare spots and all. It was not like that when I grew up, back then there were no bare spots until way into May. -- herder 8*

*Summers* were perceived to have changed the least but the herders experienced increased and shifting vegetation from lichen to grass and shrubs and a rapidly climbing tree-line.

In terms of weather conditions, the interviewees experienced and emphasized an increased variability in the weather, making it more unpredictable and unstable. Changes in the weather result in the need to move the herd and more frequent changes hence markedly increase the workload on the herders. Rapid and extreme fluctuations in temperatures and extreme weather were perceived to have become more common and several herders emphasized the increased variability and instability but they also talked about increased weather intensity.

*It’s like this (demonstrates huge waves with the hand) up and down all the time. In recent years, we’ve also started to say that when we’ve had a cold snap of say 15 degrees (°C), that’s been a lot. And we know that it’s now 2 days later and it’s above zero. This is what it’s been like in recent years. … One evening it was suddenly plus two degrees (°C). It went like from 20 to 2 degrees (°C) in just a few hours, but then it went back down again. This kind of uneven temperature is something that you think has started to occur more recently—these sudden shifts in temperature I mean. -- herder 5*

The herders had heard and learned about climate change effects from the media as well as being informed by the authorities about projected changes. Some reindeer herders even perceived projections of future climate change as an even heavier burden than the already tangible effects they had themselves noticed. As pastoralists, the reindeer herders are experts on adaptation and handling change but now articulated that they were coming under severe stress. The herders did not recognize themselves anymore and believed that the old rules of traditional knowledge no longer hold true, as the lived experiences of the older generations become less relevant for today’s herders facing what they perceive as a different weather regime.

## Quantitative study results

- O1: The snow season is beginning later and ending sooner.

Three variables were examined to explore changes in the timing and length of the snow season: its start date, end date, and duration. Across all three of these variables, the sign of the trends in the meteorological data was almost unanimously consistent with the perceptions reported by the interview subjects (Table 2). There were 10, 9, and 9 stations, respectively, where the trend in the variables was consistent with the notion of a snow season that is arriving later, ending sooner, and becoming shorter overall.

**Table 2:** Linear regression trend, percent of the variance in the time series explained, and *p* value of the trend line, for the start date, end date, and duration of the snow season at each station in the study region, 1978–2007

	Snow season start date			Snow season end date			Snow season duuration		
	Trend (days/year)	Variance explained (%)	*p* value	Trend (days/year)	Variance explained (%)	*p* value	Trend (days/year)	Variance explained (%)	*p* value
ARJ	0.46	1.7	0.578	0.59	18.2	0.060	0.13	0.1	0.893
DVD	0.18	0.1	0.882	− 0.73	7.9	0.244	− 0.91	2.4	0.528
FRO	*1.37*	*29.4*	*0.005*	*− 0.63*	*16.2*	*0.046*	*− 2.01*	*29.8*	*0.005*
GAD	*0.99*	*14.5*	*0.050*	− 0.21	4.2	0.303	*− 1.20*	*16.7*	*0.034*
GUN	*1.48*	*15.3*	*0.044*	− 0.70	18.3	0.026	*− 2.18*	*20.1*	*0.019*
JUN	*1.04*	*19.6*	*0.027*	− 0.33	5.5	0.260	*− 1.38*	*19.9*	*0.025*
KVK	*1.08*	*16.8*	*0.030*	− 0.27	8.6	0.130	*− 1.35*	*19.2*	*0.020*
LUL	*1.25*	*18.4*	*0.026*	− 0.14	5.1	0.258	*− 1.39*	*19.6*	*0.021*
PAJ	0.07	0.1	0.882	− 0.28	9.1	0.111	− 0.35	1.3	0.559
STO	0.09	0.5	0.729	− 0.06	0.3	0.789	− 0.15	0.7	0.673

Statistically significant trends (increasing or decreasing, *p* < 0.05) are highlighted with italic text

**Fig. 2:**
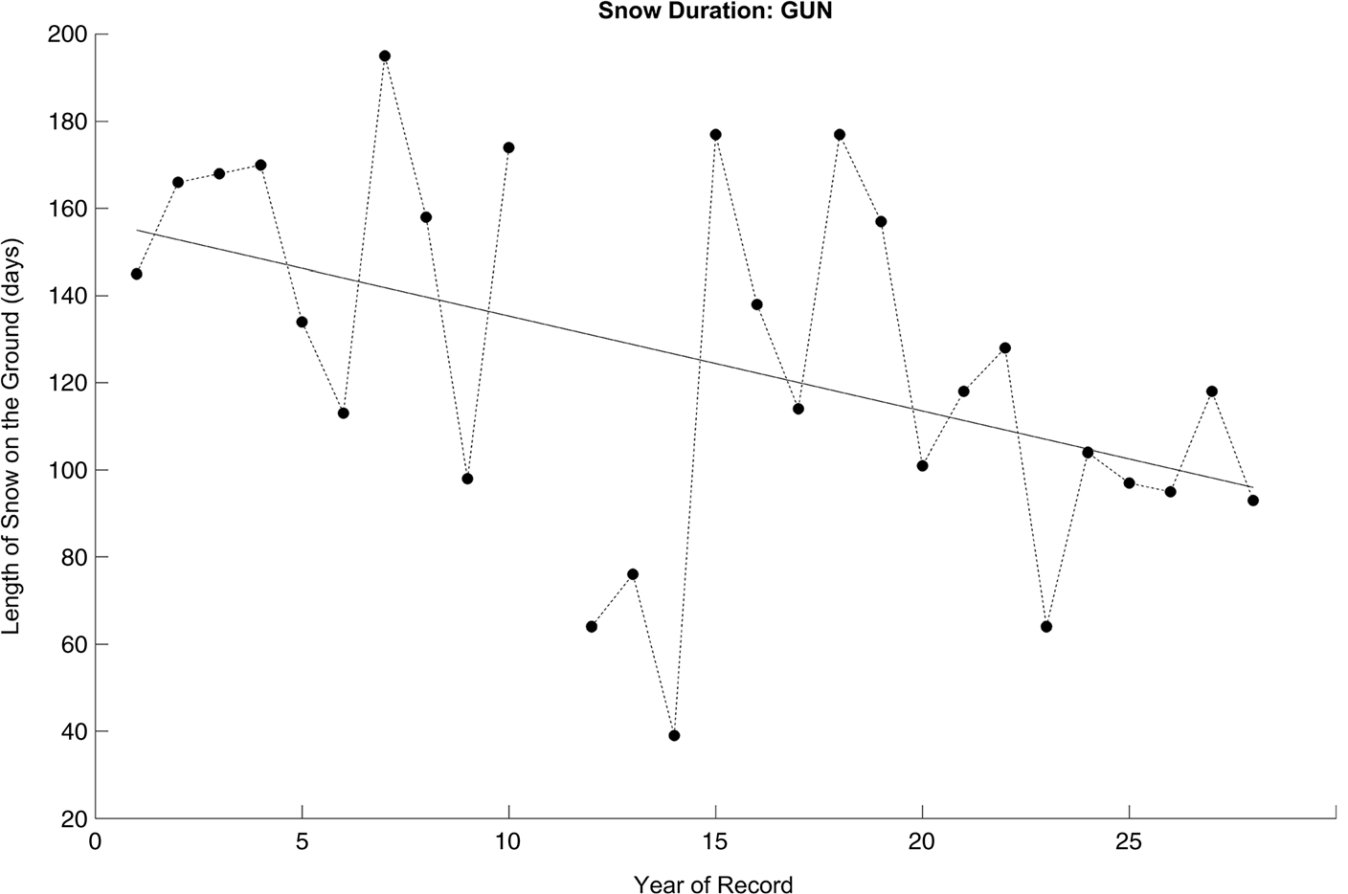
Annual count of the length of the season with 25 mm or more of snow cover observed consecutively at Gunnarn, 1978–2007. The solid line indicates the statistically significantly (*p* < 0.05) best-fit least-squares linear regression trend. Note: Snow cover observations were incomplete for winter 1988-1989 at this station; this year was assigned a missing value for regression analysis

Despite the consistency in the sign of the trends to the perceptions, statistically significant trends were only evident at roughly half of the stations examined. At Gunnarn and Froson, the only stations with a statistically significant trend in all three variables, the regression model for snow season duration suggested a decrease of more than 2 days per year over the study period (Fig. 2). This trend indicates that the snow season in recent years is approaching two full months shorter than it was in the late 1970s given our definition of consecutive days with 25 mm or more of snow cover.

- O2: Wintertime temperatures are increasing.

**Fig. 3:**
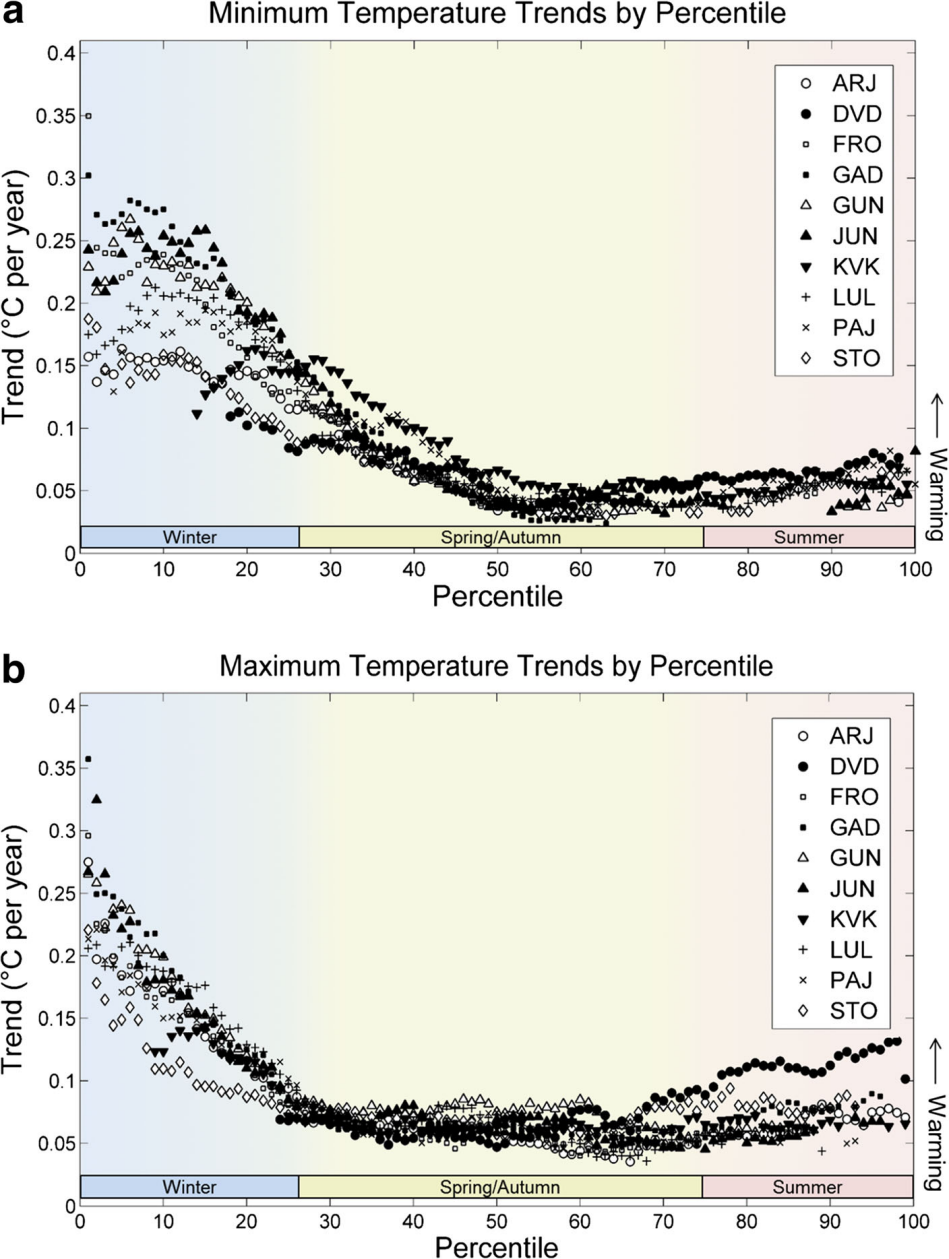
Trends in each percentile of daily minimum (a) and maximum (b) temperature at each station in the study region, 1978–2007. Only statistically significant trends (*p* < 0.05) are shown on the plot. Color bars on the bottom of the figure indicate approximate segments of the temperature distribution corresponding to each season of the year

There was robust evidence in the meteorological record that wintertime temperatures have increased during the lifespan of the interviewed subjects in the region they inhabit. Across the study region, most percentiles of temperature were found to be increasing throughout the time period examined, and in many cases, these trends were statistically significant (Fig. 3a, b). There were no instances of statistically significant decline in temperature at any station or percentile examined out of the 2000 tests performed.

Wintertime temperatures—those associated with the lowest percentiles of annual temperatures—were observed to be increasing more rapidly than temperatures during other times of the year. For minimum temperatures, the increase at some stations exceeded 0.1 °C per year, and many stations had increases above 0.05 °C per year across temperature percentiles associated with the winter months. The trend across low percentiles of maximum temperature was less pronounced than for minimum temperatures, but again, many stations had increases above 0.05 °C per year. For comparison, the *global* warming trend over the period 1951–2012 (for all months of the year) was approximately 0.02 °C per year [30].

- O3: Long stable cold periods are becoming less common.

**Fig. 4:**
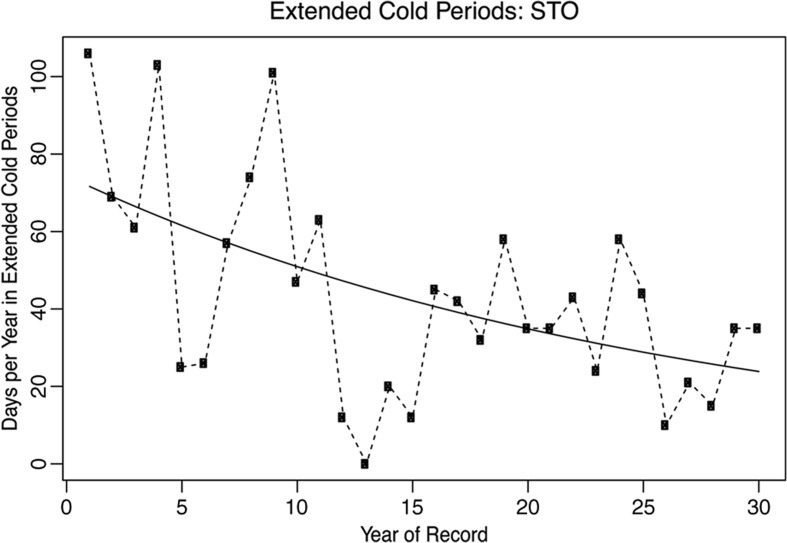
Time series of the number of days per year classified as parts of extended cold periods at Storlien, 1978–2007. The solid line indicates a statistically significant trend for a log-linear regression model relating time to the number of extended cold period days per year

There was robust evidence in support of the perception that long stable cold periods are becoming less common. The number of days per year in extended cold periods was found to be statistically significantly declining at 9 of the 10 stations in the study region, and the sign of the trend was consistent with a decline at all 10 (Table 3). At some stations, such as Storlien, it was not uncommon for more than 60 days, and sometimes more than 100 days, per year to have been classified as parts of extended cold periods in the early portion of the record (Fig. 4). Over the most recent decade, almost every year has had fewer than 50 days meeting the cold period criteria.

- O4: Rapid fluctuations in temperature are becoming more common.

**Table 3:** Slope and statistical significance of Poisson (log-linear) regression models of the trend in the number of days per year in extended cold periods at study stations, 1978–2007

	Days per year in extended cold periods	
	Trend log (days/year)	*p* value
ARJ	*− 0.031*	*0.009*
DVD	− 0.012	0.424
FRO	*− 0.037*	*0.014*
GAD	*− 0.033*	*0.009*
GUN	*− 0.033*	*0.006*
JUN	*− 0.037*	*0.004*
KVK	*− 0.029*	*0.039*
LUL	*− 0.035*	*0.010*
PAJ	*− 0.024*	*0.019*
STO	*− 0.038*	*0.002*

Rows in the table with statistically significant trends (*p* < 0.05) are highlighted in italics

**Fig. 5:**
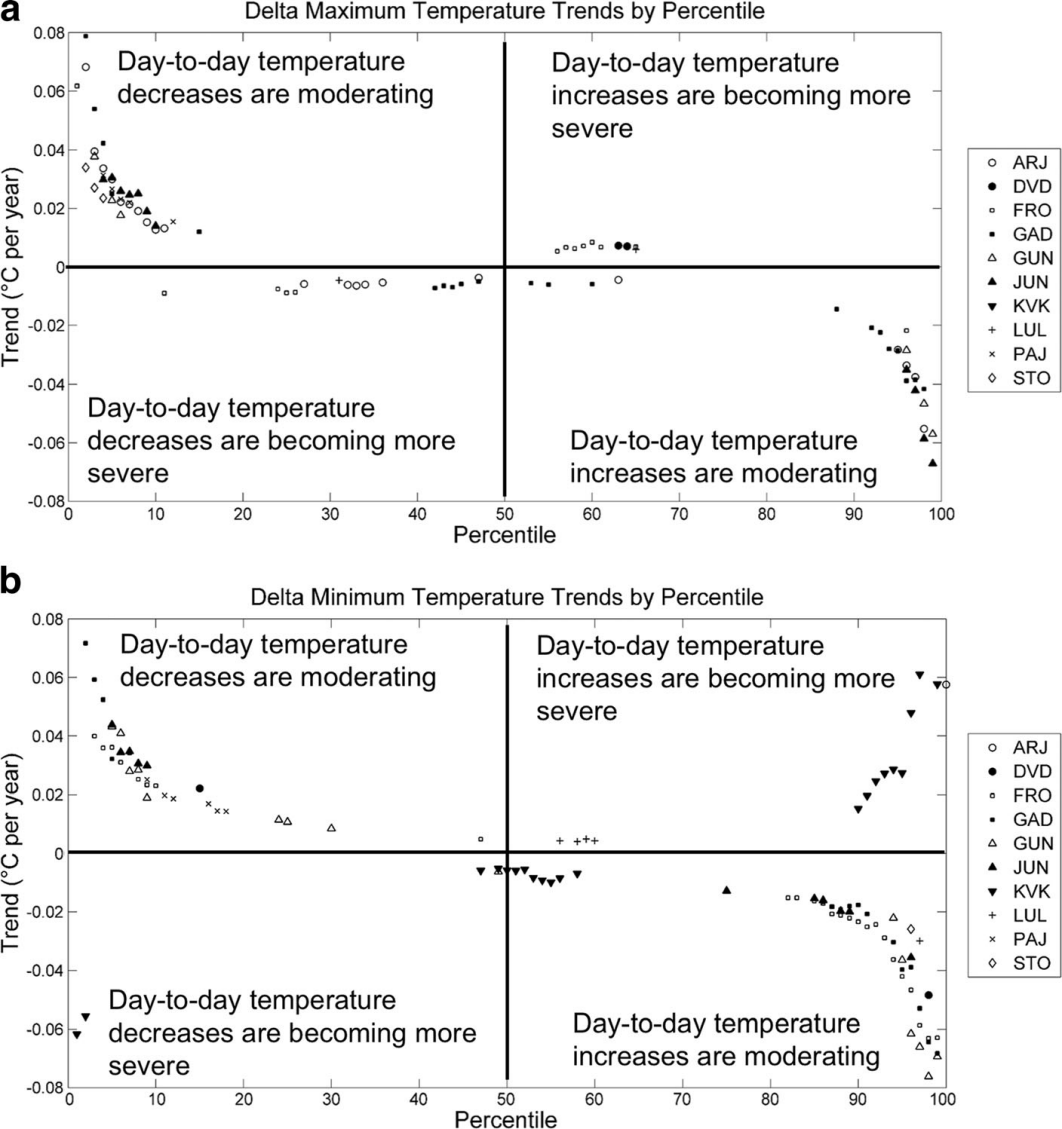
Trends in each percentile of day-to-day changes in minimum (a) and maximum (b) temperature. Only statistically significant trends (*p* < 0.05) are shown on the plot. For reference, the 100th percentile corresponds to the largest observed temperature increase from one day to the next, whereas the 1stpercentile corresponds to the largest observed temperature decrease from one day to the text

The quantitative analysis did not reveal evidence of increasingly frequent or intense rapid fluctuations in temperature using multiple methodological approaches. There were isolated instances of significant trends in day-to-day changes in minimum and maximum temperatures, and the majority of the significant trends were associated with reductions in day-to-day temperature changes (Fig. 5a, b). Only at one station, Kvikkjokk, a statistically significant increase in large day-to-day temperature changes was observed and these trends were limited to minimum temperature only. A more common result was that the largest day-to-day swings in temperature, in either the positive or negative direction, were becoming more moderate over time.

Analysis of the range in temperatures observed over 5-day periods did also not yield evidence that rapid fluctuations in temperature have become more common (Table 4). During most months of the year, none of the stations in the study region had significant increases in the 50th, 75th, 90th, or 99th percentile of 5-day temperature range. More stations were associated with decreasing 5-day temperature ranges and these decreases were almost entirely observed in the winter months.

**Table 4:** Counts of the number of stations in the study region (out of 10) where the 5-day temperature range statistically significantly increased (lefthand column) or decreased (right hand columns) within each calendar month, 1978–2007. Counts are shown for four specific percentiles of 5-day temperature range

	Number of stations with increasing ranges				Number of stations with decreasing ranges			
	Median	75th Percentile	90th Percentile	99th Percentile	Median	75th Percentile	90th Percentile	99th Percentile
Jan	0	0	0	0	*6*	*2*	0	0
Feb	0	0	*1*	0	*1*	0	0	0
Mar	1	0	0	0	0	0	0	0
Apr	0	0	*1*	*1*	0	1	0	0
May	0	0	0	0	0	0	0	0
Jun	0	0	0	0	0	0	0	0
Jul	*1*	0	0	0	0	0	0	0
Aug	*2*	*1*	*2*	*3*	0	0	0	0
Sep	*1*	*4*	*2*	*2*	0	0	0	0
Oct	0	0	0	0	0	0	0	0
Nov	0	0	0	0	*2*	*4*	*1*	*2*
Dec	0	0	0	0	*8*	*6*	*3*	*2*

All cases where at least one station reported a statistically significant trend are shown in italics

There were more stations with increasing counts of days with temperatures fluctuating near the freezing point than those with decreasing counts. A statistically significant increase in near-freezing days was detected at 2 of the 10 study stations using filters of ± 1 and 2 °C, and one station with a significant increase with a filter of ± 3 °C. The slope of the trend was positive but not significant at 5–8 stations depending on the filter used. A summary of the observations, the created variables, the methods to test them, and the outcomes are visualized in (Table 5).

**Table 5:** Summary of observations and representative quotes of the Sami reindeer herding population that were used to formulate the quantitative stand of the project, along with the specific metrics used in the quantitative analysis and subsequent results

Observations	Illustrative quotes	Quantitative test	Result
O1. The snow season is beginning later and ending sooner. Snow seasons are shorter	“And already in March the spring starts and in April with bare spots and everything. It wasn’t like that when I grew up, back then there were no bare spots until way into may”	Start date, end date, and duration of snow season	Claim supported in terms of the sign of the trend, statistical significance weak. In 28 of 30 test performances, the sign of the trend was consistent with perceptions, 14 of 30 trends were significant
O2. Wintertime temperatures are increasing	“... The winters feel much warmer”	Changes in temperature distributions	Claim strongly supported including statistically significant trends in winter temperature percentiles
O3. Long, stable, cold periods are becoming less common	“According to the interviewees, the long stable cold periods often do not occur at all ...”	Number of days each year in cold events (mean temperature < 25th percentile) lasting 10 days or more	Claim strongly supported. Claim strongly supported. 9 of 10 stations show statistically significant declines, trend is negative at all 10
O4. Rapid fluctuations in temperature are becoming more common	“It went from like − 20 to + 20 °C in just a few hours, but then it went back down again. This kind of uneven temperatures is something that you thing has started to occur more recently ...”	Day-to day changes and temperature fluctuations. 5-day temperature range. Numbers of days with temperatures fluctuating around freezing	Claim not supported. No evidence of increasing variability in the case of day-to-day changes of 5-day temperature range. Slight decrease in variability observed at some locations Generally increasing trend in days with temperatures near freezing, but few stations with significant trend

### Meta-inferences from the connected qualitative and quantitative results

In summary, this study illustrates how the winters of northern Sweden have undergone substantial changes during the last three decades and how these specific changes affect reindeer herding. Climate change effects in northern Sweden were investigated through Sami reindeer herder observations combined with 30 years of regional meteorological data. Three out of four tested changes observed for many years by the reindeer herders were consistent with the instrumental record. The contradictory result regarding observation 4—rapid fluctuations in temperature are becoming more common—could indicate that the two methods employed here did not manage to measure the same component of climate change. The concept of “weather variability” is broader than some of the more specific language used in the interviews by the Sami people about temperature patterns and timing and cold spells. Accordingly, there was the least confidence that the quantitative metric matched the perceived trends for this variable out of the four that were tested.

- O1: The snow season is beginning later and ending sooner.

The weather data provided support for the herder notion of a shorter snow cover season and data for two stations exhibited an incredible two full months decrease in snow cover over the investigated 30-year period of time. The snow season grew shorter by more than a full day every year for several stations yet the trend was not significant for all stations examined or all test examined. One station even exhibits a *later* snow season end date (ARJ). Some herders report a strong spring snow melt delay in the high mountains in a certain area while the overall herder impression was a much earlier spring onset and snow melt. The herders who report a later high mountain snow melt have winter grazing areas in the coastal region corresponding to a weather station with a strong signal of shorter snow cover (LUL). This illustrates that the combination of several changes in different directions of different local or regional areas can work together, further increasing the effect on reindeer herding. If the winter pastures become destroyed a month earlier than usual at the same time as the summer grazing lands stay unfit longer than before, then the already difficult period in the spring including the calving season gets prolonged several weeks with potentially detrimental consequences for the herders and the reindeer.

- O2: Wintertime temperatures are increasing.

All herders perceived the winters to be warmer and summers to have changed the least and there was also a more rapid increase in wintertime compared to summertime temperatures in the weather data. There was an overall temperature distribution shift to the right on a horizontal temperature scale and a compression of the distribution curve was also found, reflective of stronger trends in wintertime temperatures than other seasons. Similarly, to the high degree of consistency across the interview subjects in their perceptions of warming winters, nearly every statistical test at every station pointed toward substantial temperature increases in the cold season.

- O3: Long stable cold periods are becoming less common.

This variable also showed a strong concordance between the two methodologies. One station experienced a 50% reduction of the days in extended cold periods over the study period, yet not all stations showed statistically significant results. The decrease in extended cold periods concurs with the pronounced decline in the coldest percentiles of minimum temperatures. This decrease in cold periods could of course also be perceived as just winter warming; however, the herders’ reports of the means of change comply with decreased cold periods and again the consequences for the herders are problematic.

- O4: Rapid fluctuations in temperature are becoming more common.

There was no evidence in support of increased temperature variability in the weather data. On the contrary, a weak signal of the opposite was observed; a decrease in temperature variability, using two of the statistical approaches. Temperature or weather variability more generally may represent an aspect of climate that is perceived based on a combination of multiple various factors interacting with one another, but difficult to measure with the significant-change-in-one-parameter statistical analyses approach. The interacting factors could involve physical and psychological dimensions that were unable to be measured holistically. Tests of the number of days with temperatures fluctuating around zero revealed positive trends at most stations, although significant trends were only observed at one or two depending on the test parameters. It is possible that a change in the climate more related to key biophysical thresholds like the freezing point is what is being perceived by herders when they report increasing fluctuations because the systems on which they live and depend are highly sensitive to those thresholds. Our analysis supports this possibility, additional probing would be required to reach a strong conclusion.

## Discussion and conclusions

### Major findings

We undertook a mixed methods analysis to learn if and how community perceptions of climatic changes could complement quantitative assessments of trends in temperature and precipitation. Our objective was to use a methodological approach encompassing the complexity, subjectivity, and context-dependent high sensitivity usually associated with qualitative methods; along with the scale, consistency, generalizability, and validity more generally associated with quantitative methods (Agrawal 1995). We sought to create such insights in the context of the northern Sweden Sami reindeer herding populations’ experience to produce a clearer, more societally relevant understanding of how the climate is changing, may change in the future, and what the impacts to people and environment may be in a distinctive and rapidly changing location.

Our analysis revealed statistically significant trends for a majority of the variables tested related to climate change in northern Sweden. The results support the hypothesis that meteorological data support local perceptions. This is an important finding because there is a limited pool of evidence comparing local perceptions to meteorological records regarding climate change, as well as traditional to western knowledge, and we find a fair degree of correspondence between the two. Knowledge of climate change and its impacts can extend beyond the spatial domains covered by meteorological sensors. In places where meteorological sensors and people are collocated, mixed methods research can reveal a richer understanding of the specific mechanisms by which climate change affects society. We suggest that this is especially true for perceptions derived from indigenous peoples. In places where no meteorological sensors exist, the traditional knowledge of local people may provide equally useful information. Other scholars have noted that indigenous peoples are particularly sensitive to environmental changes and may even notice important changes before reported in the formal scientific literature (Greene et al. 2001; Krupnik & Jolly 2002; Riedlinger & Berkes 2001). The lived experience of the indigenous peoples and the inherited knowledge gained over generations from a subsistence lifestyle can provide an excellent early indicator of environmental change. Berkes and Berkes concluded that “Indigenous knowledge appears to bring some unique advantages in dealing with multiple variables and complexity” and described Indigenous peoples’ holistic way of integrating multiple complex parameters as a resemblance of a fuzzy logic system (Berkes & Berkes 2009). The greater magnitude of the reported perceived changes in the qualitative data as compared to the effect sizes described in the quantitative data may indicate such increased sensitivity among the Swedish reindeer herding Sami as well.

We also found that the extent to which local perceptions and meteorological observations aligned was dependent on the specific climatic phenomenon we examined. This is an important finding for future mixed methods research regarding climate change and its impacts. Future scholars could work to develop appropriate quantitative indicators of concepts like “climate variability” and “unpredictability” that were widely perceived as important to study participants but not apparent in the meteorological record as we examined it. Similarly, future research could also strive to explore stressors beyond climate change that may be leading to certain perceptions about how climate is affecting livelihoods.

### Insights into local climate change and its effects

Our analysis revealed dramatic changes in some climatological variables that mirrored perceptions of stress and susceptibility among interviewees. For example, we found a shortening of the snow cover season by almost two full months over a 30-year period. Similarly, we observed a decline in the number of days in extended cold periods approaching 50% at some stations. These modes of change are significant for reindeer herding. Interviewees described how the decrease in extended cold periods result in the need for maintained preparedness to move the herd and thereby increase the workload. An absence of snow also increase the workload and later formation of lake and river ice hamper and delay the annual migration in the spring and fall. The affected freeze-thaw cycle reduces accessible grazing and improves conditions for predators through the subsequent dispersion of the herds, thereby further increasing the workload as well as the reindeer herding trades’ susceptibility to predator pressure (Furberg et al. 2011). The timing and variance of weather and climate patterns are of particular importance. In polar regions, studies have shown that the observed decrease in snow cover extent and duration is mostly derived from higher temperatures in the spring months resulting in earlier and more rapid snow melt (Derksen & Brown 2011), which has differential societal impacts than if large-scale changes were observed in the fall.

Warming in the Sami region is occurring at an exceptionally rapid rate compared to other places around the globe and the warming is mainly concentrated during the winters (Derksen & Brown 2011; IPCC 2013). As shown in this study, warmer winters have vast effects on the reindeer herding community but will also affect other activities, trades, and industries as well, apart from reindeer herding. Winter sport tourism is a major activity in northern Sweden, skiing both downhill and cross-country as well as snowmobile driving or dog sledding. In a study of the future for Swedish downhill skiing published 2007 using climate change scenarios for 2070 to 2100, researchers estimated the loss of expenditure of a projected 2 months shortening of the snow cover season to be larger than the year 2006 total ski-ticket turnover of the whole Swedish ski industry (Moen & Fredman 2007). The current study has shown that several stations already exhibit a snow cover season shortening of this magnitude, as compared to the 1970s.

Nearly half of the interview subjects commented on their perceived increase in temperature variability. This perception of a shift toward greater temperature variability has been reported by many Arctic as well as non-Arctic Indigenous populations worldwide. In a study of residents of Finland and the Russian peninsula, the interview subjects used terms like “rapid,” “no stability,” “fluctuates,” “drastic,” and “apocalypse” to describe changes they had seen in temperature variability. As an example, here is a quote from reindeer herder for life, now retired, Heikki Hirvasvuopio, in Snowchange (Helander-Renvall & Mustonen 2004):

*Today, we can have almost 30 degrees of variation in a very small time frame. In the olden days, the Sami would have considered this almost like an apocalypse if similar drastic changes had taken place so rapidly.*

In light of the current study’s apparent contradiction between a perceived increase in variability and an observed decline in meteorological data variability, future work might consider metrics other than those investigated in this analysis as a means of capturing “relevant” variability to which people are sensitive (Walsh et al. 2005). We cannot be fully confident that the metrics derived to explore the notion of variability in this study are consistent with those reported by interview subjects; scientific and community interpretations of this concept may be quite different. Indigenous peoples in many locations across the world also emphatically report increased weather unpredictability (Berner et al. 2005; Fox 1998; Jolly et al. 2002; McDonald et al. 1997). This particular phenomenon merits further investigation since it has massive impacts on the subsistence lifestyles and it might represent the same—or another, not yet monitored—climate change effect as the increased temperature variability.

In this study, we have provided a new analysis of temperature trends in the region by examining individual percentiles and visualizing those trends in the context of temperature distribution shifts. In the Intergovernmental Panel on Climate Change Fourth Assessment Report (IPCC AR4), there is some discussion of how future temperature distributions might compare to those of today. Some global climate models suggest a shift in the mean value toward higher temperatures and a growing tail at the high end of the distribution, such that record-setting and extreme heat days will increase in frequency at a greater rate than overall temperature increases (Christoph et al. 2004). IPCC AR5 revisited this discussion and reported that, to date, it is unclear if there are substantive changes occurring to the global temperature distribution beyond a shift in the mean. This study did not find any shifts indicative that an increase in the variance of the temperature distribution has occurred in this study region over the past three decades. Instead, the results show a shift toward generally higher temperatures accompanied by a slight compression of the temperature distribution. The entire distribution is shifting to the right, but the left portion of the distribution (associated with low winter temperatures) is moving at a greater rate (see Supplemental Fig. 1). This pattern is different from the asymmetrical trends and implied increased variability reported for 100 stations across Europe since 1975 (Klein Tank & Können 2003), but our study is focusing on a much smaller geographic region.

Some of the specific shifts in temperature distribution that have occurred in northern Sweden are particularly challenging for reindeer herding and the effects of these specific changes become visible through the qualitative data. To facilitate effective adaptation measures, the need for more detailed information together with an interpretation of the expected effects by local experts, in this case, the reindeer herders, is key for success. More detailed weather data monitoring with higher spatial resolution is essential to be able to handle and adapt to current and future change. Most of the planning for adaptation has so far had a national focus; however, people experience climate change impacts at the local scale and the localized effects of climate change vary greatly from place to place. This geographical variability underlines the need for local action both in terms of vulnerability assessments and in terms of the development of adaptation strategies (Measham et al. 2011). Local information on indigenous peoples’ experiences, perceptions, and knowledge is necessary to capture, as a basis to select the right adaptation strategies to increase the overall resilience of local communities. This study contributes to the limited research body that claims policy makers should more explicitly consider indigenous peoples’ reports on perceptions of climate change as a valued resource when developing adaptation policies, plans, and strategies. Given the changes already reported in the scientific literature, we encourage policy makers in the Arctic reindeer herding regions to implement and support national adaptation policies including action plans that offer a sustainable future for reindeer herding. Examples of societal adaptation measures of great importance for the reindeer herders could be secured grazing areas with preserved old forests along migration paths and the provision of safe modes of passage across water reservoirs. If the changing characteristics of winters favor predators, as reported by our interviewees, then taking this fact into account when deciding upon predator population sizes could be another powerful policy measure to support sustainable reindeer herding. The herders’ experience and expertise in adaptation may also help and guide adaptation in other areas of the society.

### Perspectives on mixed methods climate change research

The impacts of climate change and peoples’ perceptions of climate change might have important public health consequences through causal pathways even more complex than those described elsewhere in this manuscript. For example, public media have covered an increased number of suicides among young male Sami. The researcher AO Eikeland has reported about suicide and mental illness as an Arctic problem (Eikeland 1999). Scholars have reported several underlying causes behind the mental health problems and climate change is one of them (Cunsolo Willox et al. 2015). Our present study clearly shows how climate change poses a threat to the reindeer herding identity and lifestyle. JP Stoor et al. have hypothesized that suicide for Sami in Sweden might have become a way to preserve one’s Sami identity when the identity is being threatened, for example, if one must give up reindeer herding (Stoor et al. 2015). In this context, climate changes then pose direct threats to life. The Lancet Commission on health and climate change published 2015, reported on mental health impacts and anxiety-related responses from climate change through different pathways and about solastalgia (Albrecht et al. 2007), a sense of loss experienced when peoples’ land had been damaged and they had lost resources (Watts et al. 2015). There are a few existing studies on climate change and mental health in the Arctic but there is a need for further research efforts in this context (Cunsolo Willox et al. 2012).

The development of scientific evidence by its nature takes time, whereas peoples’ perceptions of climate change in relation to indigenous knowledge may provide an early signal of change. It has been reported that in small island communities, hydro-meteorological hazards have been precisely predicted by local people, based on their observations of changes in the environment, celestial bodies, and behavior of animals, in relation to the time of the year they happen. These observations were also considered being indicators of other hazards (Hiwasaki et al. 2014). Humans living close to nature observe changes before data analysis reveals statistically significant trends. Research methodology needs to be continuously developed to show complex and elusive phenomena, and mixed methods may capture what exists “between” methods that neither of the methods would have captured if used on their own.

Limitations of this study include classical objections to mixed methods work such as the possibility of not investigating the same phenomena, for example, weather variability, with the two different methodologies. Additionally, from a statistical perspective, this study at first glance committed serious violations with respect to the multiple comparison problem, increasing the probability of making a type I error (Saville 1990). The focus in this study, however, is not on any individual regression slope or statistical measure. Instead, we focused on the broad patterns and effect sizes and signs along with statistical tests. Although we performed many tests without any adjustment for the *p* value, the multiple comparison problem should not confound the results.

To conclude, this study of climate change effects in northern Sweden illustrates the complementary nature of mixed methods research by demonstrating some of the major changes this region is experiencing through two different lenses and how these changes affect reindeer herding. Increasing temperatures, especially wintertime, along with a decrease in extended cold periods and the snow cover season, are among a suite of social, environmental, and economic stressors that continue to impact the lifestyle the Sami reindeer herders—largely to their detriment. This analysis has demonstrated that, in many cases, the perception of those who most closely interact with the environment provides an accurate representation of climate change as measured by a network of meteorological stations. As such, the local perception of climate change can and should be important in shaping policy and the voice of advocates and policymakers.

## Electronic supplementary material

Fig. S1 (PNG 436 kb)

High Resolution Image (TIF 487 kb)

Fig. S2 (PNG 443 kb)

High Resolution Image (TIF 492 kb)

## Footnotes

1 The objective of the Swedish predator policy, regulated in law, is to preserve and sustain viable populations over time including target numbers of bears (*Ursus arctos*), wolves (*Canis lupus*), eagles (*Aquila chrysaetos*), wolverines (*Gulo gulo*), and lynx (*Lynx lynx*).There is a system in place to compensate reindeer husbandry losses; however, it is considered gravely insufficient by reindeer herder organizations. The estimated number of reindeer lost to predators are 19,500 to 82,500 reindeers, every year. (SOU 2012:22)
