# Supplementary figures and images for: In the light of change: a mixed methods investigation of climate perceptions and the instrumental record in northern Sweden
Source: Popul Environ. 2018 Aug 28;40(1):47–71. doi: 10.1007/s11111-018-0302-x (PMC6132962; doi:10.1007/s11111-018-0302-x)

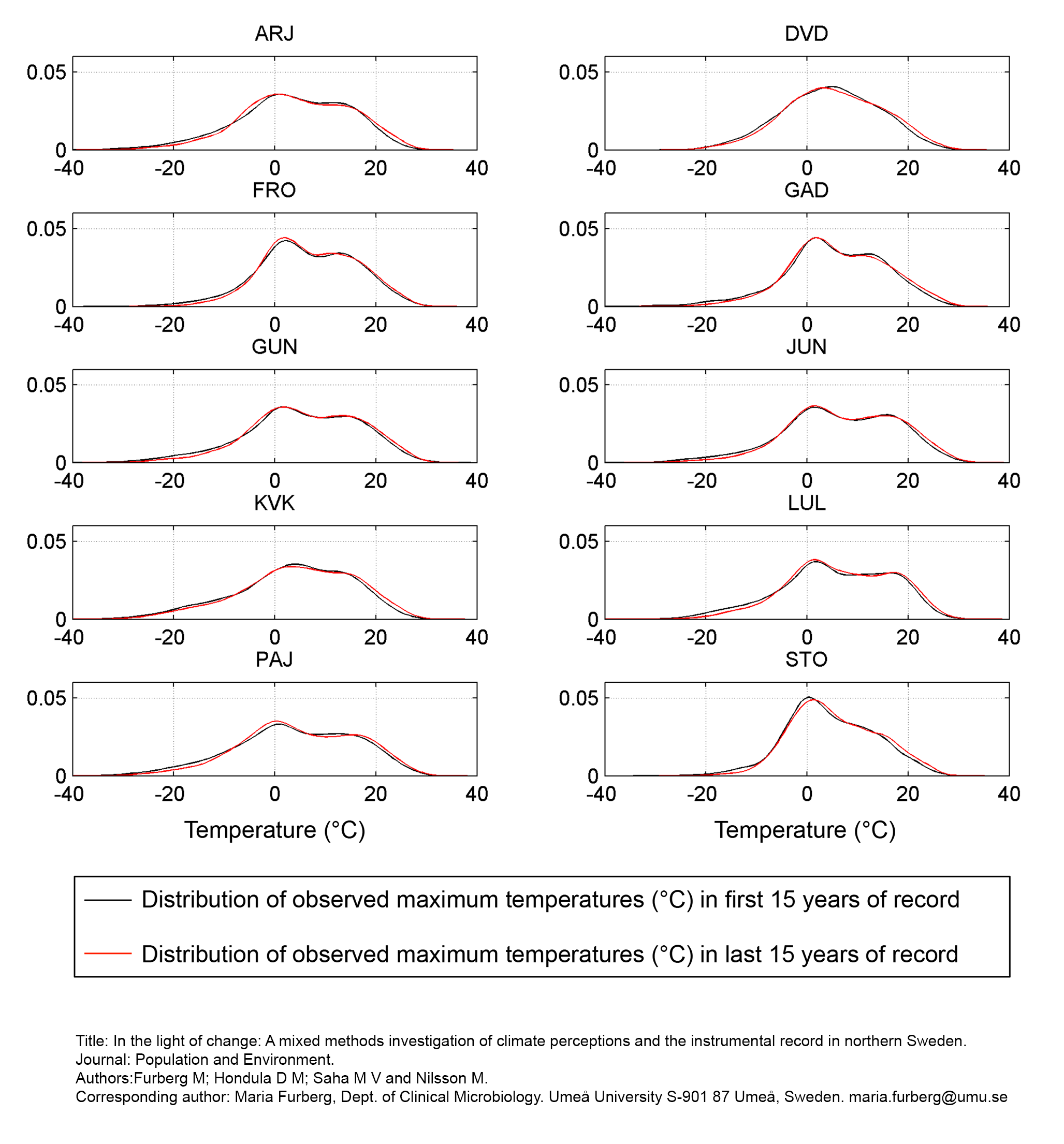

Supplement: Supplementary file 1 — (PNG 436 kb) [file 11111_2018_302_Fig6_ESM.png]

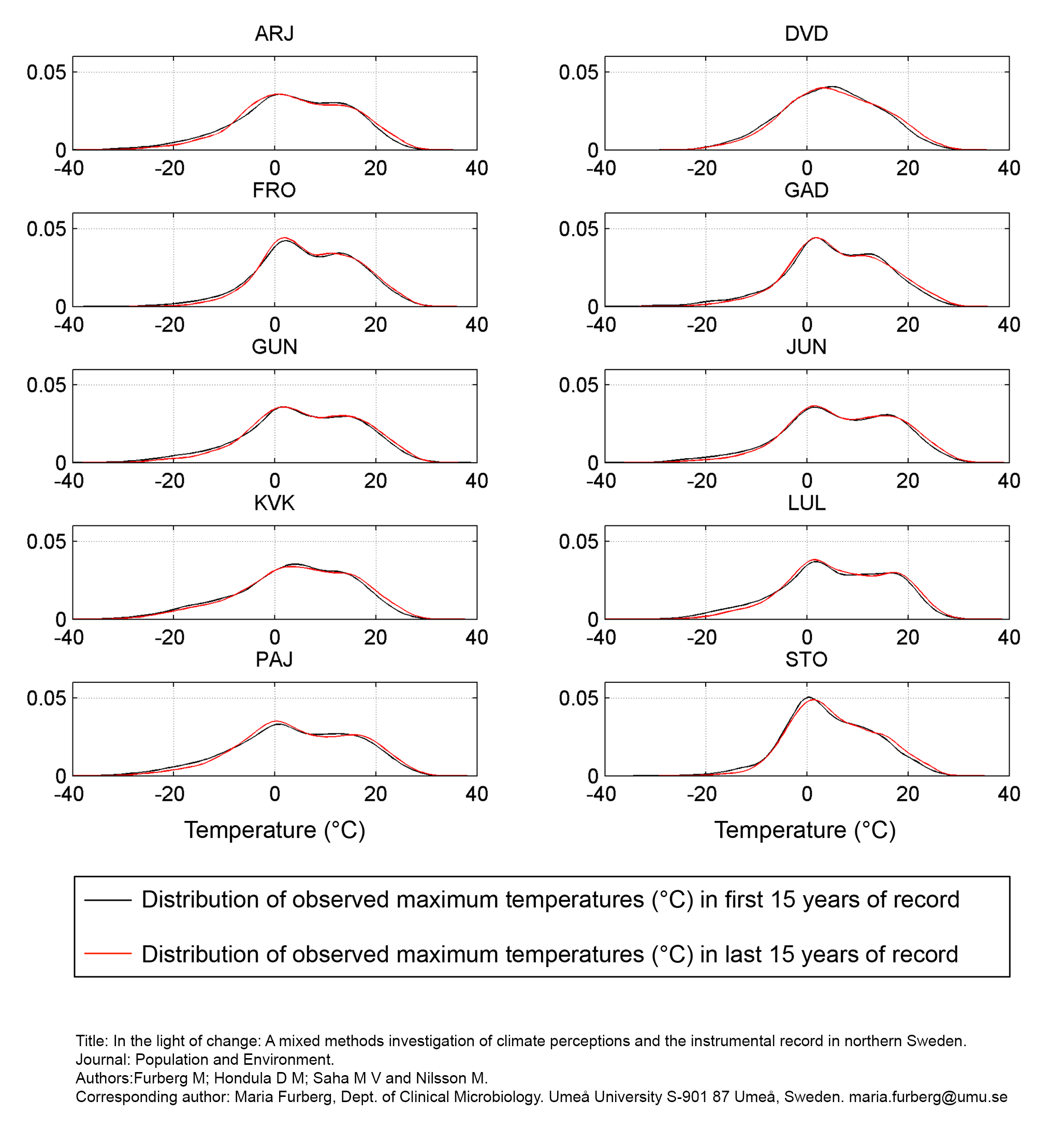

Supplement: Supplementary file 2 — High Resolution Image (TIF 487 kb) [file 11111_2018_302_MOESM1_ESM.tif]

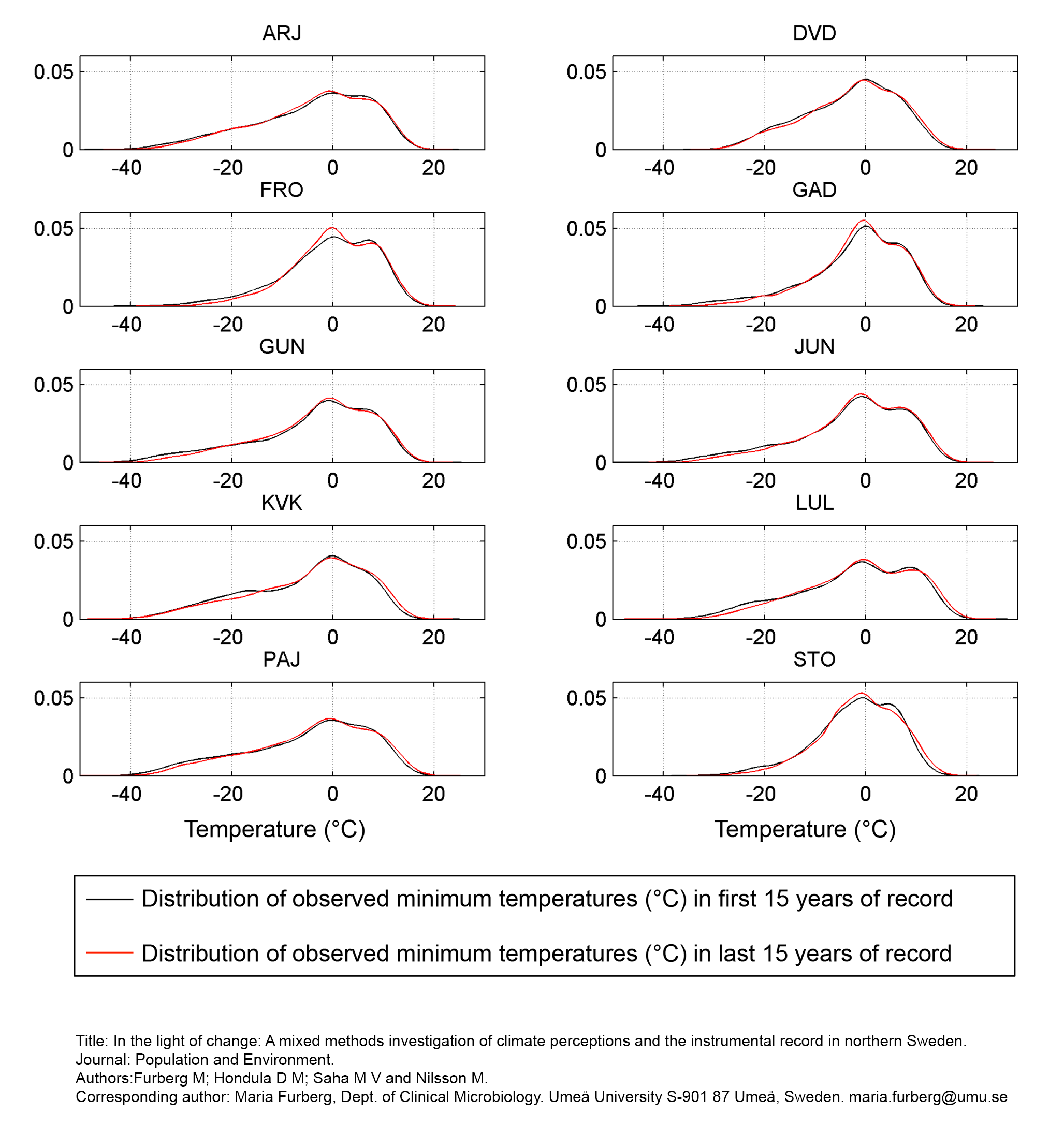

Supplement: Supplementary file 3 — (PNG 443 kb) [file 11111_2018_302_Fig7_ESM.png]

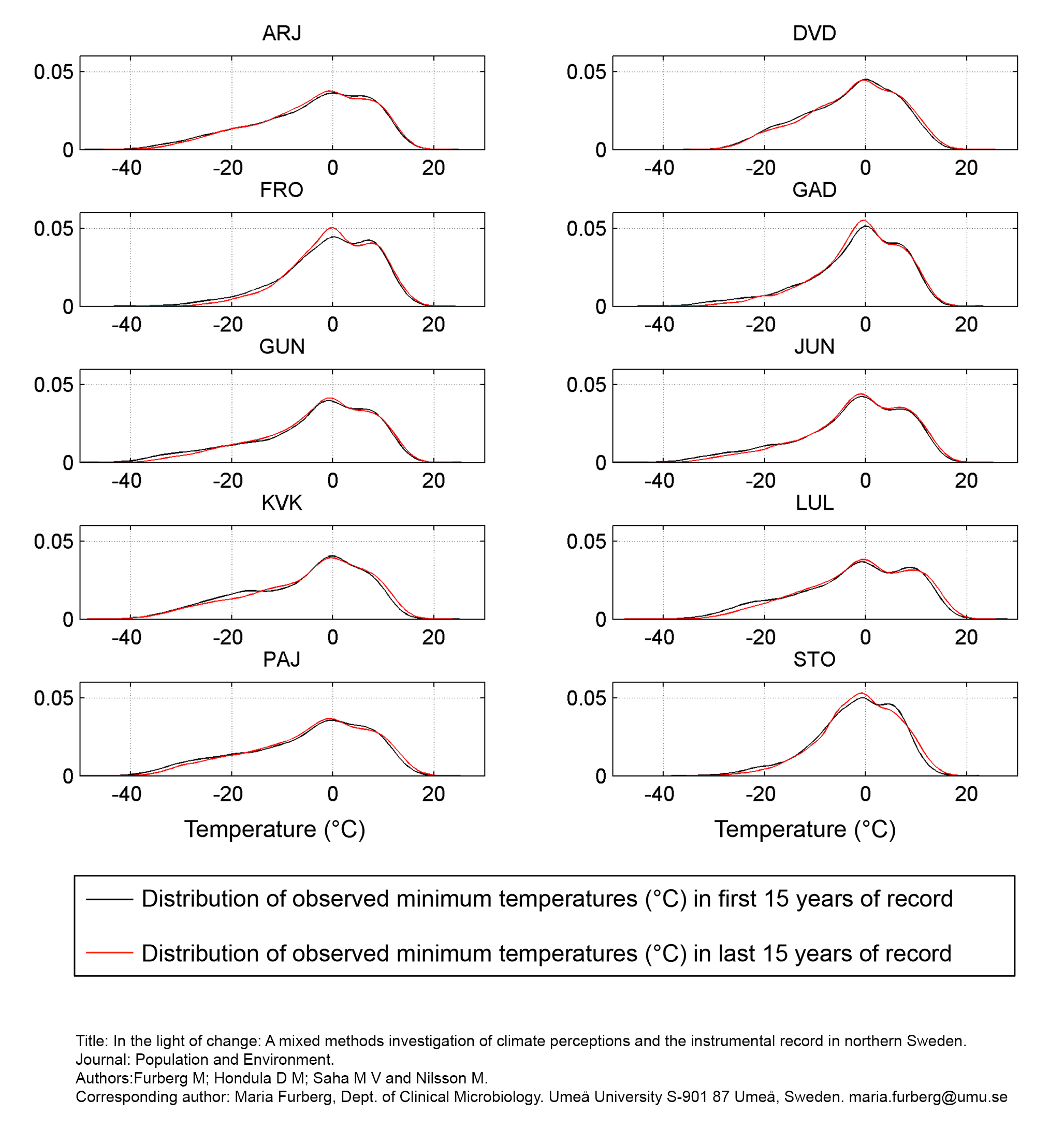

Supplement: Supplementary file 4 — High Resolution Image (TIF 492 kb) [file 11111_2018_302_MOESM2_ESM.tif]
